# Role of Oxidative Stress in Modulating Unfolded Protein Response Activity in Chronic Myeloid Leukemia Cell Line

**DOI:** 10.7508/ibj.2016.01.009

**Published:** 2016-01

**Authors:** Ali Bazi, Mohammad Reza Keramati, Mehran Gholamin

**Affiliations:** 1 Cancer Molecular Pathology Research Center, Imam Reza Hospital, Faculty of Medicine, Mashhad University of Medical Sciences, Mashhad, Iran;; 2 Faculty of Allied Medical Sciences, Zabol University of Medical Sciences, Zabol, Iran;; 3 Division of Human Genetics, Immunology Research Center, Avicenna Research Institute, Mashhad University of Medical Sciences, Mashhad, Iran

**Keywords:** Unfolded protein response, Oxidative stress, Endoplasmic reticulum stress

## Abstract

**Background:**

Recently, it has been revealed that tyrosine kinase inhibitors (TKIs) act through inducing both oxidative and endoplasmic reticulum (ER) stress in chronic myeloid leukemia cells. However, ER stress signaling triggers both apoptotic and survival processes within cells. Nevertheless, mechanisms by which TKIs avoid the pro-survival effects are not clear. The aim of this study was to evaluate the potential role of oxidative stress in activity of unfolded protein response (UPR) survival pathway within K562 cell line.

**Methods:**

The expression of UPR survival target genes, Xbp1, and Grp94 (glucose requiring protein 94) was studied in single and combined exposure to oxidative and ER stress in K562 cell line by quantitative and qualitative PCR.

**Results:**

The expression of UPR-related survival gene Grp94 was hampered by exposing to oxidative stress in cell induced with ER stress.

**Conclusion:**

Interaction of oxidative and ER stress may role as a mediator influencing UPR signaling activity.

## INTRODUCTION

Chronic myeloid leukemia (CML) is a common hematologic malignancy. Pathophysiologic features of CML arise from uncontrolled enzymatic activity of a fusion protein named break point cluster region-Abelson (BCR-ABL)^[^^1^^]^. Current conventional treatment for CML includes tyrosine kinase inhibitor (TKI) agents^[^^2^^,^^3^^]^. Recent studies have demonstrated that anti-cancer agents, including TKIs, exploit oxidative stress as a participating mechanism in their therapeutic effects^[^^4^^,^^5^^]^. 

Recently, it has been indicated that endoplasmic reticulum (ER) stress-induced signaling cascade known as unfolded protein response (UPR) participates in CML cells as one of the mechanisms employed by TKI to trigger apoptosis^[^^6^^-^^8^^]^. UPR is a stress signaling pathway within eukaryotic cells. This pathway employs three ER trans-membrane proteins: inositol-requiring protein, protein kinase R-like ER kinase, and activating transcription factor 6^[^^9^^,^^10^^]^. Each of these branches is involved in molecular activities, which ultimately cause either overcoming stress situation or exposing to apoptotic cell death. Inositol-requiring protein, as the main pro-survival route of UPR, induces activation of a cytoplasmic inactive transcription factor, i.e. unspliced X-box binding protein 1 (uXbp1) to its active form, spliced Xbp1 (sXbp1). Splicing process of Xbp1 includes the excision of a 42 length base from uXbp1 transcript via enzymatic activity of an inositol-requiring protein^[^^11^^]^.

Considering the role of UPR in triggering survival and apoptotic routes, survival/apoptotic counterbalance in CML cells subsequent to TKI treatment is not clear. With regard to the interrelated functions of both oxidative and ER stress on UPR activity^[^^12^^]^, we examined if oxidative stress could be a part of inhibitory mechanism involved in suppressing survival branch of UPR in K562 cell line. 

In the present study, K562 cell line was cultured in different oxidative/ER stress conditions using tunicamycin (Tm) and thapsigargin (Tg) as ER stress inducers and also using hydrogen peroxide (H_2_O_2_) as the oxidative stress inducer. Then Xbp1 and Grp94 (glucose requiring protein 94) expressions were evaluated by reverse-transcriptase and real-time PCR method. Although UPR activation in K562 cell line induced overexpression and splicing Xbp1, expression of Grp94, as the main target of sXbp1, was blocked in conditions of combinations of oxidative and ER stress. These results suggest the potential role of oxidative stress as a possible part of TKI actions in suppressing UPR-related survival branch. 

## MATERIALS AND METHODS

**Cell culture and treatments**

K562 cell line was purchased from Pasteur Institute of Iran (Tehran). RPMI medium containing 10% FBS and 1% pen-strep was prepared to cultivate cells. Cells were cultured in T-25 flaks to meet required 95% viability confirmed using Trypan-blue staining. Then 1 × 10^6^ cells were transferred to 6-well plates and exposed to different stress conditions. In control group, cells were treated with 0.1% DMSO. Seven stress categories were designed, including two individual (Tg-treated and Tm-treated) and two combinational-simultaneous (Tg + H_2_O_2_ and Tm + H_2_O_2_) treatments. Also, two groups with first four hours were exposured to oxidative (H_2_O_2_) and then to ER stress (Tg or Tm). Total period of exposure was eight hours. The utilized concentrations of H_2_O_2_, Tg, and Tm were 3 µM, 5 μg/L, and 1 µM, respectively. 

**RNA extraction and cDNA synthesis**

Total RNA extraction kit and cDNA synthesis kit were purchased from Parstous company (Iran). RNA was extracted from 1 million cells, and its quality was confirmed using 1% agarose gel electrophoresis. cDNA was synthesized according to manufacturer's instructions and confirmed using a housekeeping GAPDH gene through amplification reaction.

**Reverse-transcriptase PCR**

To assess uXbp1 and sXbp1 expressions, forward: 5-CCTTGTAGTTGAGAAC CAGG-3 and reverse: 5-GGGGCTTGGTATATATGTGG-3 primer sequences were used. Reaction mixture was prepared as follows: 1 pM mixed primer, 2 µl 10× buffer, 1.4 µl MgCl_2_ (1.5 mM), 0.3 µl Taq DNA polymerase, and 0.5 µl dNTP. Reaction was carried out by 40 cycles, followed by denaturation, annealing, and extension phases of 94ºC for 10 minutes, 60ºC for 30 seconds, 72ºC for 30 seconds, respectively. PCR products were assessed on 4% agarose gel electrophoresis to identify Xbp1 splicing and to evaluate the intensity of its expression. GAPDH was used as the internal positive control.

**Real-time PCR**

To measure the expression of sXbp1 target gene, Grp94, quantitative real-time PCR procedure was applied using SYBER Green dye (Parstous, Iran). The primer sequences were used as forward: 5’- TCGCCTCAGTTTGAACATTGAC-3’ and reverse: 5’-CTTCTGCTGTCTCTTCAGGTTCTTC-3’. Termal cycles were set as an initial denaturation at 95°C (10 minutes), followed by 40 cycles of 95°C (15 seconds), 60°C for annealing (1 minute) and 72°C for extension (30 seconds). Tube mixture contained 10 µl SYBER Green dye, 1 µl primer mix (10 pmol), 1 µl cDNA, and 0.4 µl ROX dye. Reaction was carried out using a Stratagene Mx3000 instrument, and GAPDH was used as the normalizer. Results were analyzed to assess Grp94 fold changes compared to the control group using two-fold changes as cutoffs for meaningfulness changes.

**Statistical analysis**

SPSS software was applied to evaluate the data statistically. One-way ANOVA and paired-sample *t*-test was used as statistical tests.

## RESULTS

**Individual ER stress and combinational states of oxidative/ER stress induce splicing and over-expression of Xbp1**

**Fig. 1:**
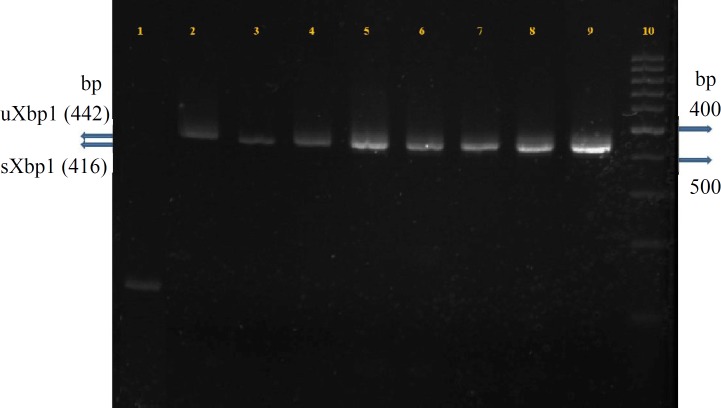
Xbp1 expression and splicing status in different individual ER stress and combinational oxidative/ER stress conditions. Line 1, GAPDH (103 bp); line 2, control (0.01% DMSO); lines 3 and 4, individuals treatments (Tg and Tm treated, respectively); lines 5-7, simultaneous combinational treatments (Tg + Tm, Tg + H_2_O_2_, Tm + H_2_O_2_, respectively); lines 8 and 9, H_2_O_2_ priority combinational treatments (H_2_O_2_ [4 h] + Tg, H_2_O_2_ [4 h] + Tm, respectively). In priority combinational groups, cells were first treated with H_2_O_2_ for four hours, and then Tg or Tm were applied for additional four hours. Line 10 represents loading 100 bp ladder. sXbp1 (416 bp) band shows increasing intensity in the range of line 1 through line 8, which represents the overexpression of spliced form in all individual and combinational stress groups. Altered splicing state of Xbp1 from unspliced (uXbp1, 442 bp) form to spliced form is the result of removing the 26-bp fragment following the activation of inositol-requiring protein branch of UPR

In this study, in both individual states of ER stress and combinational states of oxidative/ER stress, Xbp1 was overexpressed and mainly changed to its spliced (active) form. Also, sXbp1 displayed stronger expression in combinational states, which seems to be consistent with stress intensity (Fig. 1).

**Grp94 expression was significantly suppressed in combinational states of ER/oxidative stress**

Grp94 expression showed overexpression in exposure to ER stress inducers. However, in combinational states of oxidative/ER stress, Grp94 expression was suppressed to various amounts. In simultaneous combinations of oxidative stress with Tg or Tm, a mean two-fold reduction was observed in Grp94 expression compared to individual states of Tg or Tm. In contrast, such reduction in Grp94 expression was not observed in combinational state of Tg + Tm. 

**Fig. 2:**
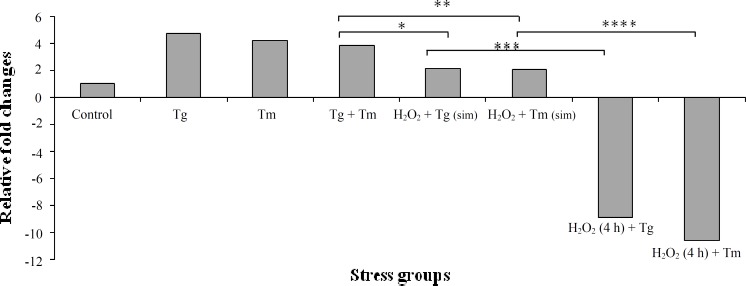
Grp94 expression in different stress conditions in comparison to the control group. Cells were exposed to individual treatments with Tg or Tm in which Grp94 expression elevated 4.7 and 4.2 folds, respectively. Interestingly, Grp94 expression showed reduction in simultaneous combinations of Tg + Tm, Tg + H_2_O_2_, and Tm + H_2_O_2_ in comparison with three first stress groups (3.8-, 2.1-, and 2-fold changes in Tg + Tm, Tg + H_2_O_2_, and Tm + H_2_O_2_, respectively). Significant reduction of Grp94 was observed in combinational groups with prior exposure to H_2_O_2_. In H_2_O_2 _(4 h) + Tg and H_2_O_2_ (4 h) + Tm situations, Grp94 expression was reduced 8.9 and 10.6 times (*P* versus ^*^0.001, ^**^0.002, ^***^ 0.007, ^****^0.006

More prominently, ER stress-induced Grp94 expression was significantly suppressed in cells initially exposed to the oxidative stress,. Figure 2 shows the fold changes of Grp94 in each stress group.

## DISCUSSION

It has been demonstrated that BCR-ABL oncogene upregulates the expression of Xbp1 and Grp78 molecular chaperon in CML cells^[^^7^^]^. It has been suggested that such activity participates as a survival mechanism recruited in neoplastic cells by this fused peptide^[^^7^^]^. BCR-ABL fused tyrosine kinase also triggers the accumulation of beta catenin peptide within cytoplasm of leukemic cell in response to TKI therapy. In turn, this phenomenon induces pro-survival UPR and enables neoplastic cells to resist against therapeutics^[^^6^^,^^13^^]^. Arsenic sulfide and resveratrol also intensify the effects of imatinib to initiate apoptosis in CML cells partly through activation of UPR pathway^[^^14^^,^^15^^]^. Furthermore, imatinib can suppress survival branch of UPR, named protein kinase R-like ER kinase; however, mechanism of this suppression in not fully understood^[^^16^^]^.

Paschen *et al.*^[^^17^^]^ reported down-regulation of Xbp1 target genes (Grp94 and Grp78) in Tg-induced neurological cell lines previously exposed to H_2_O_2_. Regarding our results, this data may suggest a role for oxidative stress in modulating UPR activity, especially in combinational states with ER stress. The effects of oxidative stress on leukemic cells have been under extent evaluations for decades; however, adverse or favor effects of oxidative stress on progression or regression of hematologic tumors still is obscure^[^^18^^]^. It seems that despite potential role of reactive oxygen species (ROS) in evolving resistant mutations in BCR-ABL fusion gene, oxidative stress exerts beneficial roles in boosting the efficiency of CML drug therapies. Not only TKIs but also other anti-cancer agents have been reported to use ROS production as a mechanism for exerting their effects in CML cells^[^^19^^-^^21^^]^. 

It has been demonstrated that oxidative stress involves in increasing cell susceptibility to apoptosis, partly through inhibiting protein kinase R-like ER kinase and pro-survival UPR pathway^[^^16^^]^. In the present study, we showed that in association with ER stress, oxidative stress can also inhibit transcriptional function of UPR-related survival transcription factor, sXbp1 which is implicated by suppressed expression of Grp94 in combinational states. Such phenomenon is likely to happen subsequent to imatinib treatment in CML patients as a result of beta-catenin cytoplasmic accumulation. However, it is probable that other mechanisms cause survival UPR to be blocked in leukemic cells, and therefore more studies are require in this area. 

Our observation highlights the role of oxidative stress as an important contributing factor in acquiring efficient results upon TKI treatment in CML. Considering unusual location of Xbp1 as a transcription factor in cytoplasm of cells, failure to induce expression of Xbp1 target gene may be due to either defective translocation of sXbp1 to nucleus or impairment trans-activating function of sXbp1 in oxidative/ER stress and combinational states in K562 cell line.
